# Supplementary material for: Cell-type and subcellular compartment-specific APEX2 proximity labeling reveals activity-dependent nuclear proteome dynamics in the striatum
Source: Nat Commun. 2021 Aug 11;12:4855. doi: 10.1038/s41467-021-25144-y (PMC8357913; doi:10.1038/s41467-021-25144-y)
Supplement: Supplementary file 1 — Supplementary Information [file 41467_2021_25144_MOESM1_ESM.pdf]

Supplementary information:

Cell-type and subcellular compartment-specific APEX2 proximity labeling reveals activity-dependent nuclear proteome dynamics in the striatum

Dumrongprechachan, V.[1,2], Salisbury, R.B.[3,4], Soto, G.[1], Kumar, M.[1], MacDonald, M.L.[3,4]*, and Kozorovitskiy, Y.[1,2]*

[1]Department of Neurobiology, Northwestern University, Evanston, IL 60208

[2]The Chemistry of Life Processes Institute, Northwestern University, Evanston, IL 60208

[3]Department of Psychiatry, University of Pittsburgh, Pittsburgh, PA 15213

[4]Biomedical Mass Spectrometry Center, University of Pittsburgh, Pittsburgh, PA 15213

*Co-corresponding authors: macdonaldml@upmc.edu, Yevgenia.Kozorovitskiy@northwestern.edu

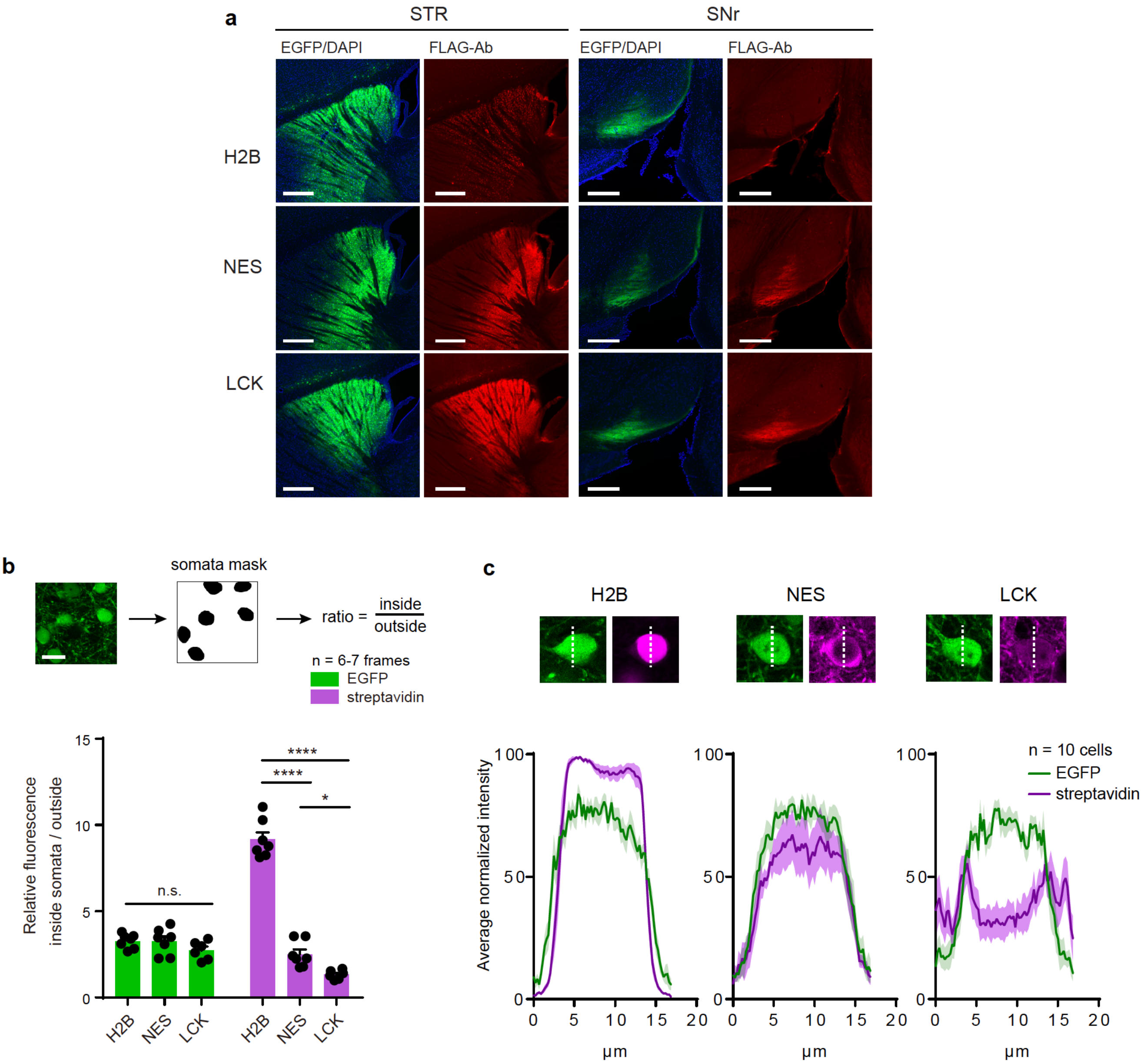

Supplementary Figure 1. Subcellular targeting of APEX constructs.

(a) Cre-dependent expression patterns of APEX variants in the striatum and the SNr. EGFP, DAPI, immunostained FLAG-Ab for APEX in green, blue, and red, respectively (scale bars: 400 µm). n = 3 animals per construct.

(b) Distribution of APEX-dependent biotinylated proteins. *Top*, an example of somatic masks used in ratio calculation for each image frame. *Bottom*, summary data for relative fluorescence signal inside and outside somata: green for EGFP, and magenta for streptavidin (n = 7 for H2B, NES, n = 6 independent regions/frames for LCK). One-way ANOVA with Tukey's multiple comparisons tests: EGFP, $F(2,17) = 1.688$, $p = 0.2145$, streptavidin, $F(2, 17) = 185.3$, $p < 0.0001$. H2B vs NES, adj. $p < 0.0001$, H2B vs LCK, adj. $p < 0.0001$, and NES vs LCK, adj. $p = 0.0435$. *$p < 0.05$, ** $p < 0.01$, *** $p < 0.001$. Scale bar: 20 µm. Error bars reflect SEM.

(c) Line scan analysis of biotinylated proteins across neuronal somata. *Top*, an example of a vertical line scan drawn across an APEX-expressing neuron. *Bottom*, fluorescent signal in the EGFP and streptavidin channels were plotted and normalized to the maximum intensity (n = 10 cells/construct). Dotted line scan: 17 µm. Shaded regions reflect SEM. Source data are provided as a Source Data file.

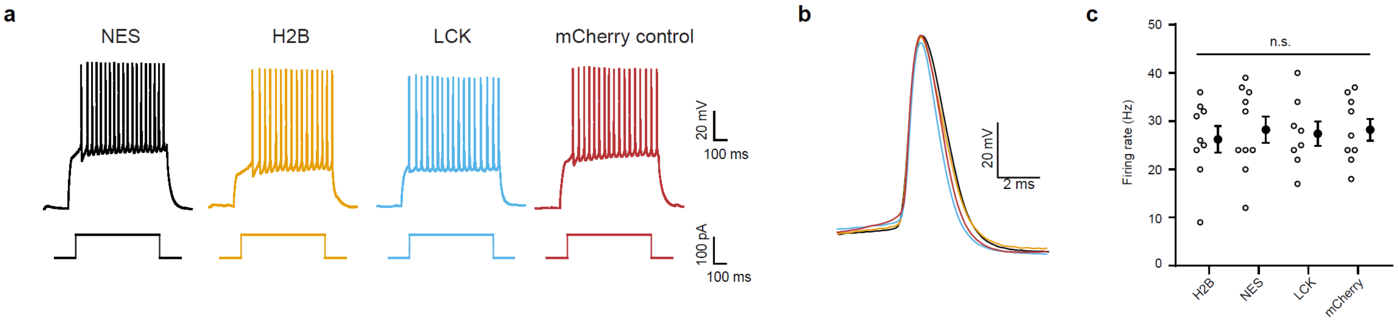

Supplementary Figure 2. Electrophysiological characterization of APEX-expressing neurons.

(a) Current-clamp recordings of APEX-expressing dSPNs.
(b) Examples of action potential shape across APEX constructs.
(c) Summary for dSPN firing rate (Hz) with 200 pA current injections. One-way ANOVA with Tukey's multiple comparisons test: $F (3, 32) = 0.1334$, $p = 0.9395$. H2B n = 9, NES n = 10, LCK n = 8, and mCherry control n = 9 cells. Summary data mean ± SEM. Source data are provided as a Source Data file.

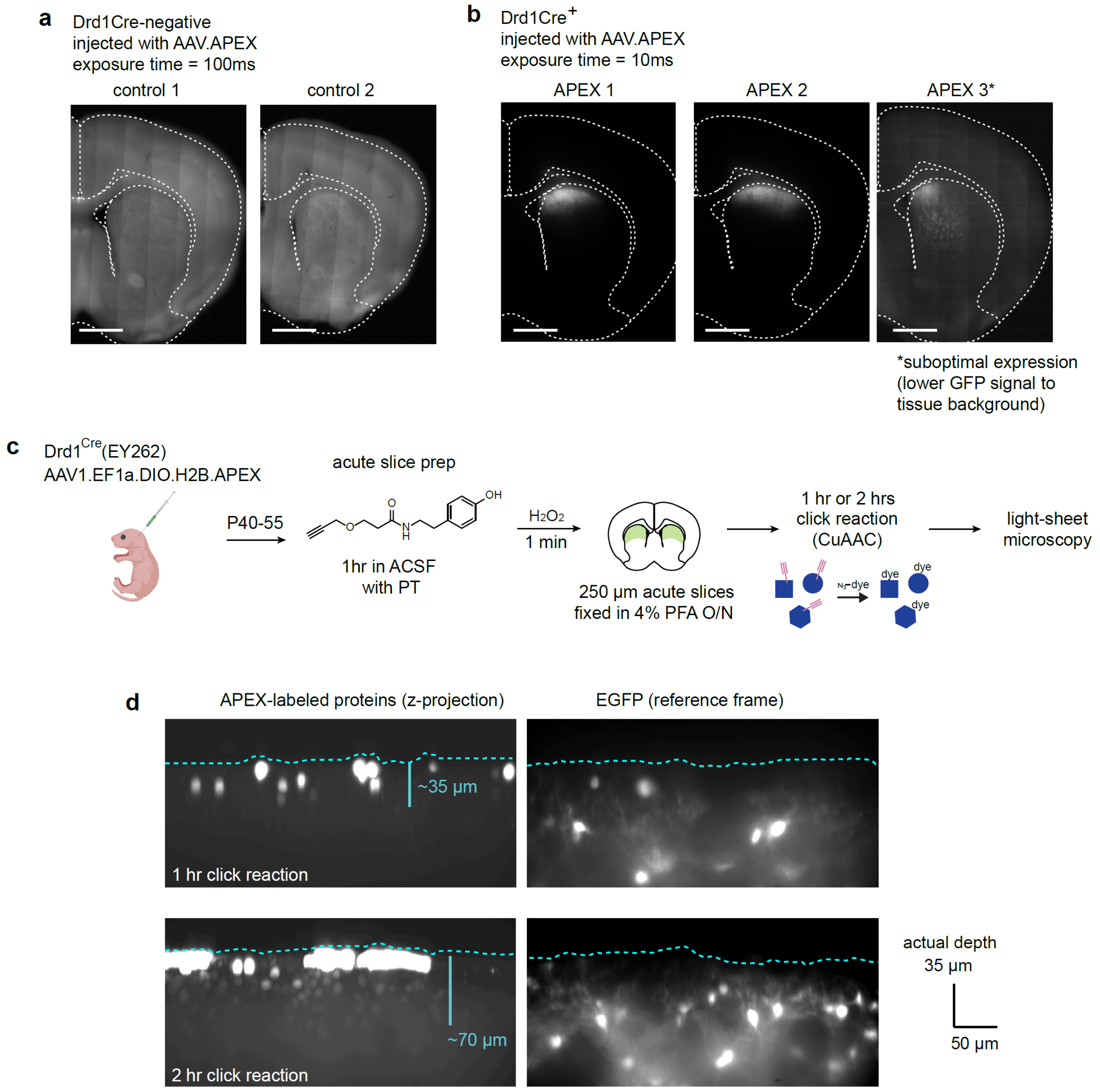

Supplementary Figure 3. Analysis of APEX expression variability and depth-dependent labeling.

(a) Examples of APEX expression variability in the acute slices. Each section represents an individual subject. Cre-negative control animals received AAV transduction but did not express APEX. Background tissue fluorescence signal was detected at 100 ms exposure.

(b) Same as (a) for Drd1^{Cre+}. Expression of APEX-EGFP+ in the dorsal striatum detected at 10 ms exposure. * denotes a subject with suboptimal expression and enhanced brightness adjustment relative to the first two (lower EGFP signal to tissue background).

(c) Workflow for APEX labeling depth analysis in the acute slices using scanning oblique plane illumination light-sheet microscopy. Acute slices were incubated in ACSF with propargyl tyramide (PT) for 1 hr. Slices were fixed with 4% PFA prior to click reaction for 1-2 hr.

(d) Cross section of acute slices imaged. PT-labeled neurons were detected as deep as ~70 µm below the surface.

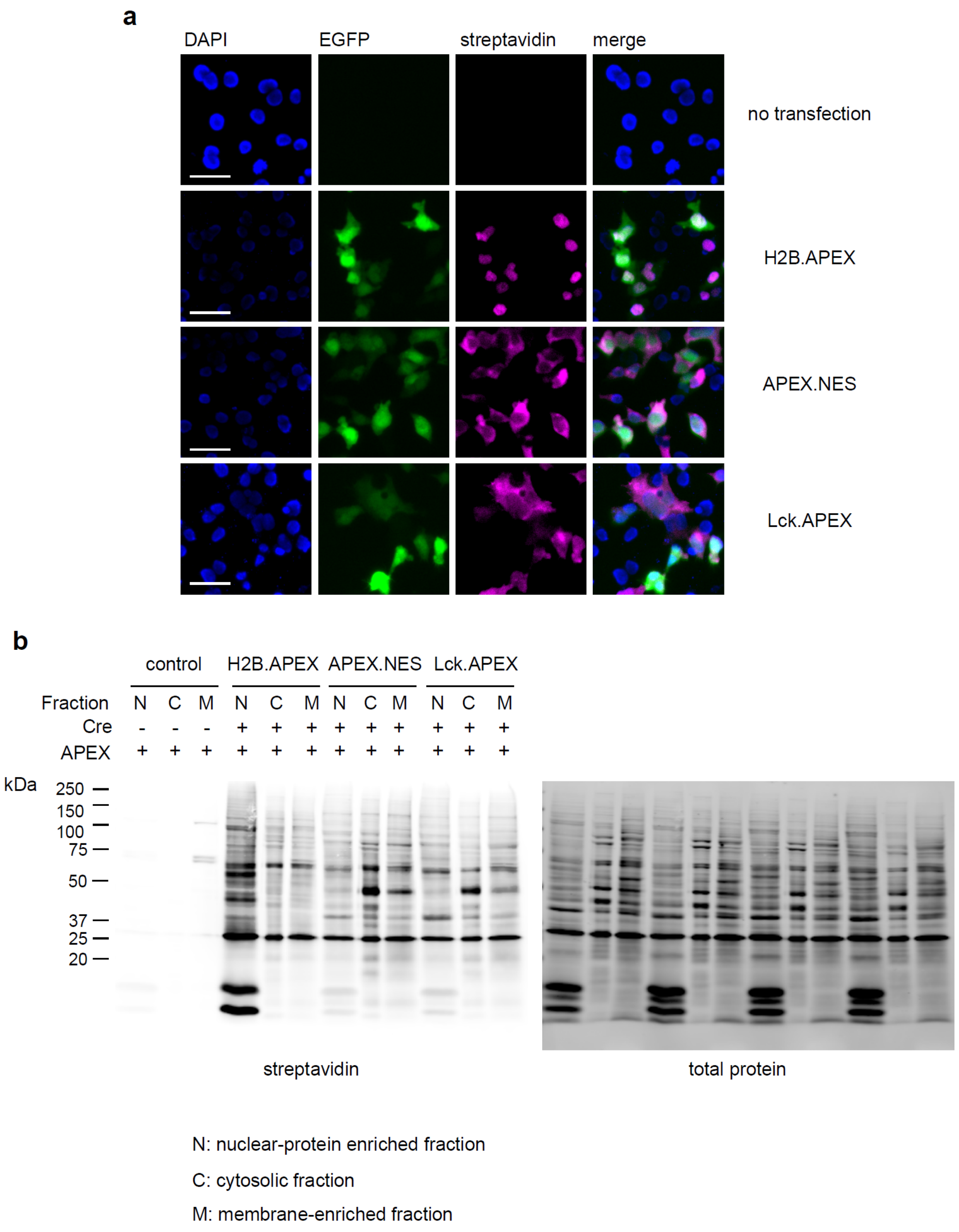

Supplementary Figure 4. Biochemical fractionation analysis of APEX activity in HEK293T.

(a) Expression and biotinylation activity of APEX constructs in HEK293T cells. Scale bar: 40 μm. n = 3 biological replicates.

(b) Western blot analysis of APEX biotinylation patterns across different subcellular fractions in HEK293T. N: nuclear fraction, C: cytosolic fraction, and M: membrane fraction. Samples were collected from multiple independent biotinylation experiments. Western blot analysis of these samples was performed once.

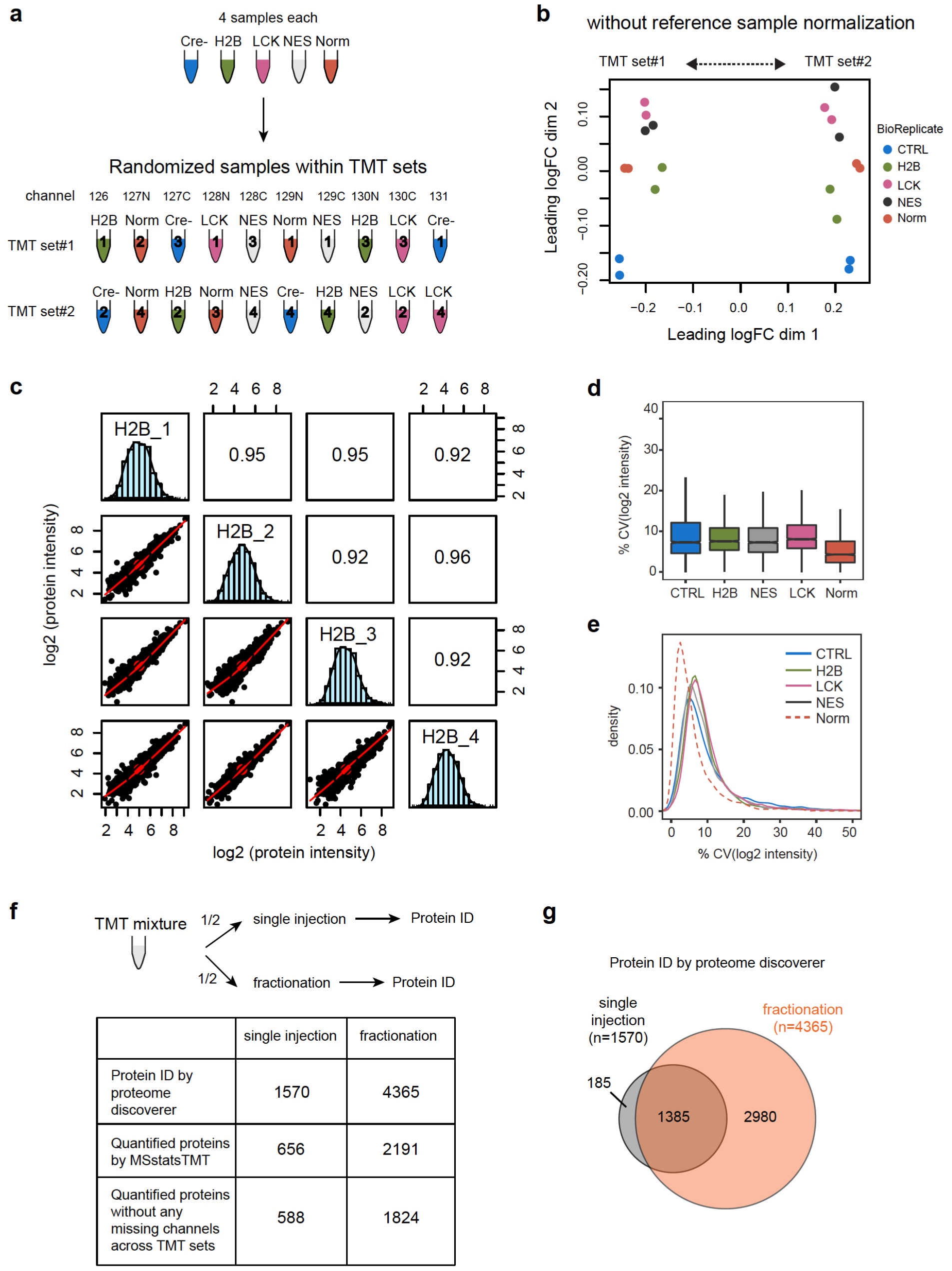

	single injection	fractionation
Protein ID by proteome discoverer	1570	4365
Quantified proteins by MSstatsTMT	656	2191
Quantified proteins without any missing channels across TMT sets	588	1824

Supplementary Figure 5. Analysis of MS sample preparation reproducibility.

(a) Experimental design schematic. Samples were randomized across two TMT 10-plex sets (top and bottom). Direction left-to-right was ordered by TMT channels (126-131). Each set contained 2 complete blocks (each block = Norm, NES, H2B, LCK, Cre-).

(b) Multidimensional clustering of log2 protein intensities without reference channel normalization. Each point was a biological replicate. Arrow indicates a clear separation between TMT sets, when reference normalization was not performed.

(c) Multiscatter plot of H2B biological replicates (example). *Diagonal*, histograms of protein log2 intensities. *Upper right corner*, Pearson correlation coefficient *r*, *Lower left corner*, pairwise scatter plots between replicates, red lines are loess-fit lines.

(d) Box plot of %CV distribution (n = 1824 proteins without missing values over n = 4 biological replicates). The upper and lower bounds of the box represent 75th and 25th percentiles while the whiskers extend from minimum to maximum. The horizontal center line is the median.

(e) Density plot of %CV distribution.

(f) Protein identification and quantification before and after high pH-reverse phase fractionation.

(g) Overlap between protein ID before and after high pH-reverse phase fractionation. Source data are provided as a Source Data file.

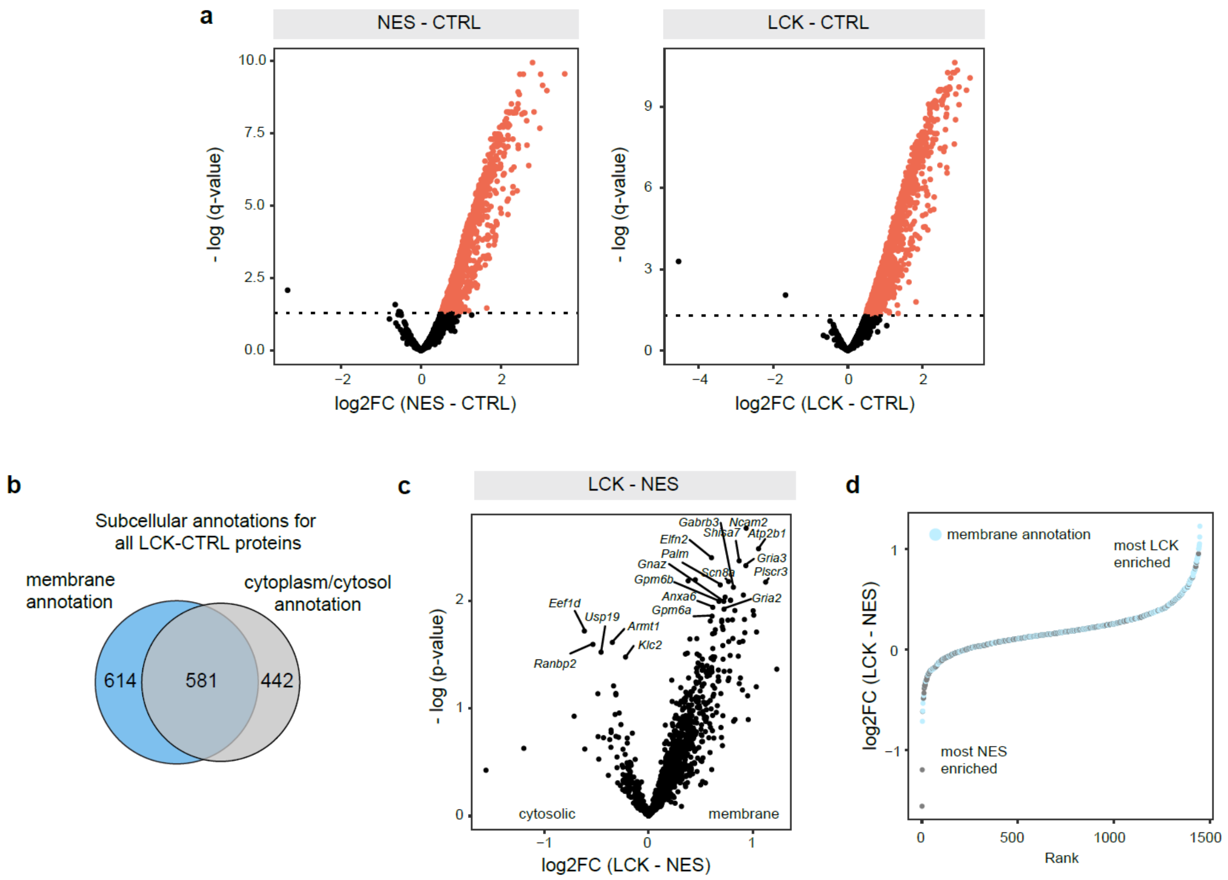

Supplementary Figure 6. Biotinylation by APEX.NES and LCK.APEX.

(a) Comparison between APEX.NES or LCK.APEX and Cre-negative control.
(b) Subcellular annotation for LCK-CTRL enriched proteins. Venn diagram shows that majority of LCK-CTRL enriched proteins have both membrane and cytoplasm/cytosol annotations.
(c) Comparison between LCK.APEX and APEX.NES.
(d) Rank plot for LCK – NES. Greater log2 (LCK– NES) ratios indicate a greater enrichment by the LCK construct. Source data are provided as a Source Data file.

a

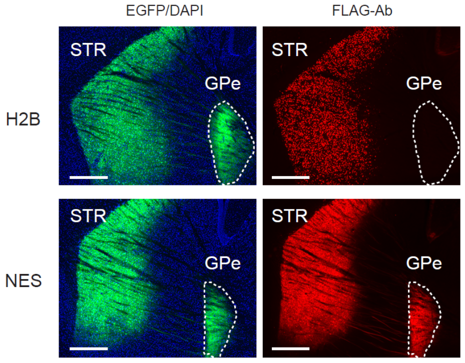

b

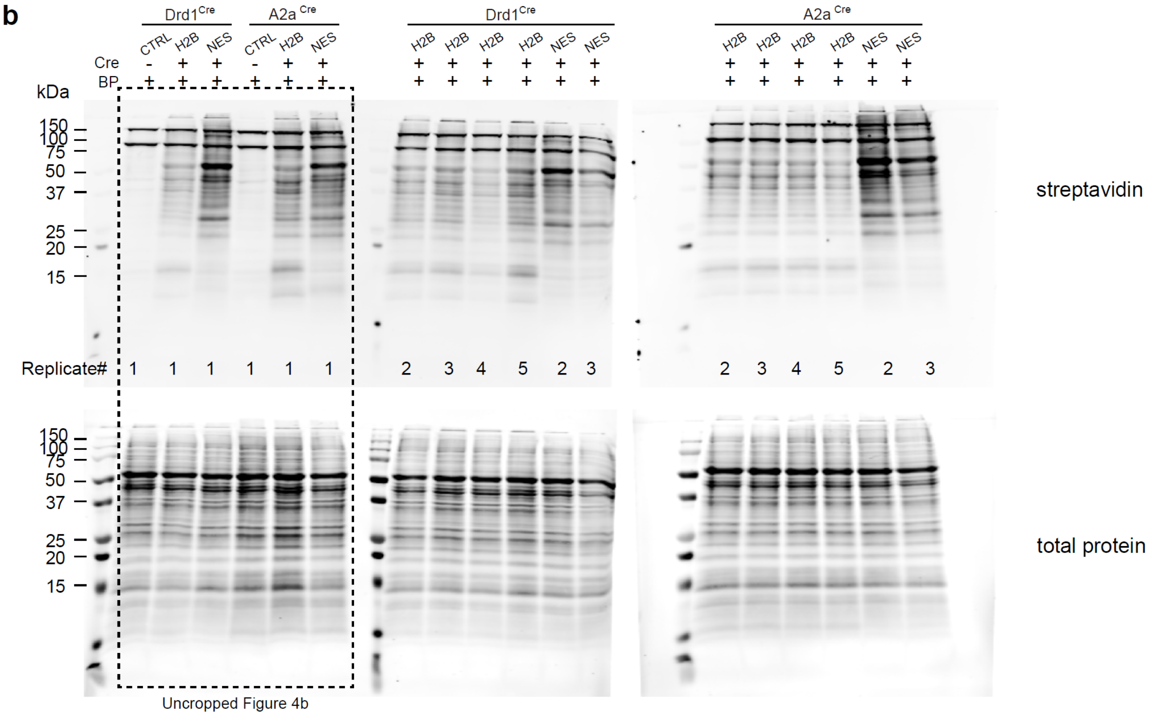

c

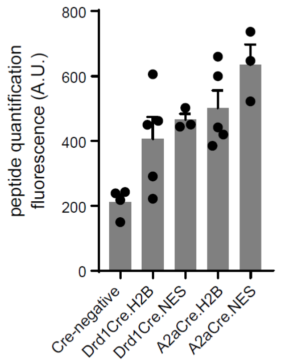

d

TMTpro 16plex experimental design

channel	126	127N	127C	128N	128C	129N	129C	130N	130C	131N	131C	132N	132C	133N	133C	134N
cell type	A2a	Drd1	A2a	A2a	Drd1	A2a	A2a	Drd1	A2a	Drd1	A2a	Drd1	Drd1	A2a	Drd1	Drd1
APEX	NES	NES	H2B	NES	H2B	H2B	H2B	H2B	NES	H2B	H2B	NES	H2B	H2B	H2B	NES
replicate#	1	3	4	3	5	2	1	1	2	2	3	1	4	5	3	2

condition	No. of replicates
Drd1.H2B	5
Drd1.NES	3
A2a.H2B	5
A2a.NES	3

Supplementary Figure 7. Characterization of APEX expression in striatal A2a^{Cre+} neurons and experimental design for SPN comparison.

(a) Cre-dependent expression patterns of APEX variants in the striatum and the GPe of the A2a^{Cre} mouse line. EGFP, DAPI, immunostained FLAG-Ab for APEX in green, blue, and red, respectively. Scale bar: 0.5 mm. n = 2 animals per construct.
(b) Western blot analysis of biotinylated lysates before MS sample preparation. Dotted line shows cropped region for blot in Fig 4b. Western blot analysis of these samples was performed once prior to orthogonal confirmation by proteomics.
(c) Relative peptide output after on-bead digestion before TMT labeling. Cre-negative control n = 4, Drd1^{Cre}.H2B n = 5, Drd1^{Cre}.NES n = 3, A2a^{Cre}.H2B n = 5, and A2a^{Cre}.NES = 3 animals/sample. Error bars reflect SEM. Source data are provided as a Source Data file.
(d) TMTpro 16plex experimental design.

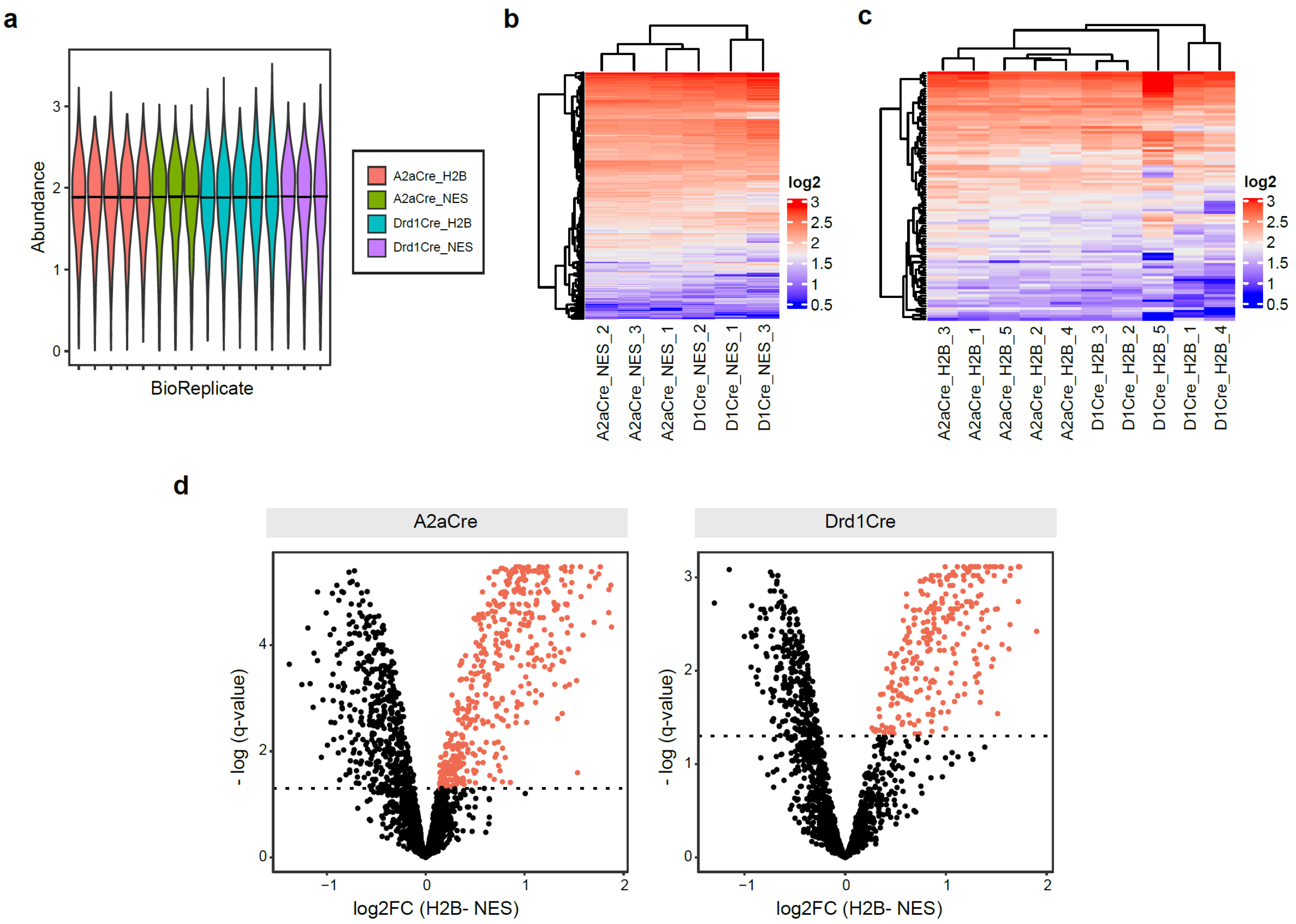

Supplementary Figure 8. Nuclear proteome cutoff analysis for dSPNs and iSPNs.

(a) Overall protein intensities across biological replicates after protein-level median normalization.
(b) Hierarchical clustering for APEX.NES samples.
(c) Hierarchical clustering for H2B.APEX samples.
(d) H2B-NES comparison in A2a^{Cre} and Drd1^{Cre} samples. Red dots indicate H2B-enriched proteins. Source data are provided as a Source Data file.

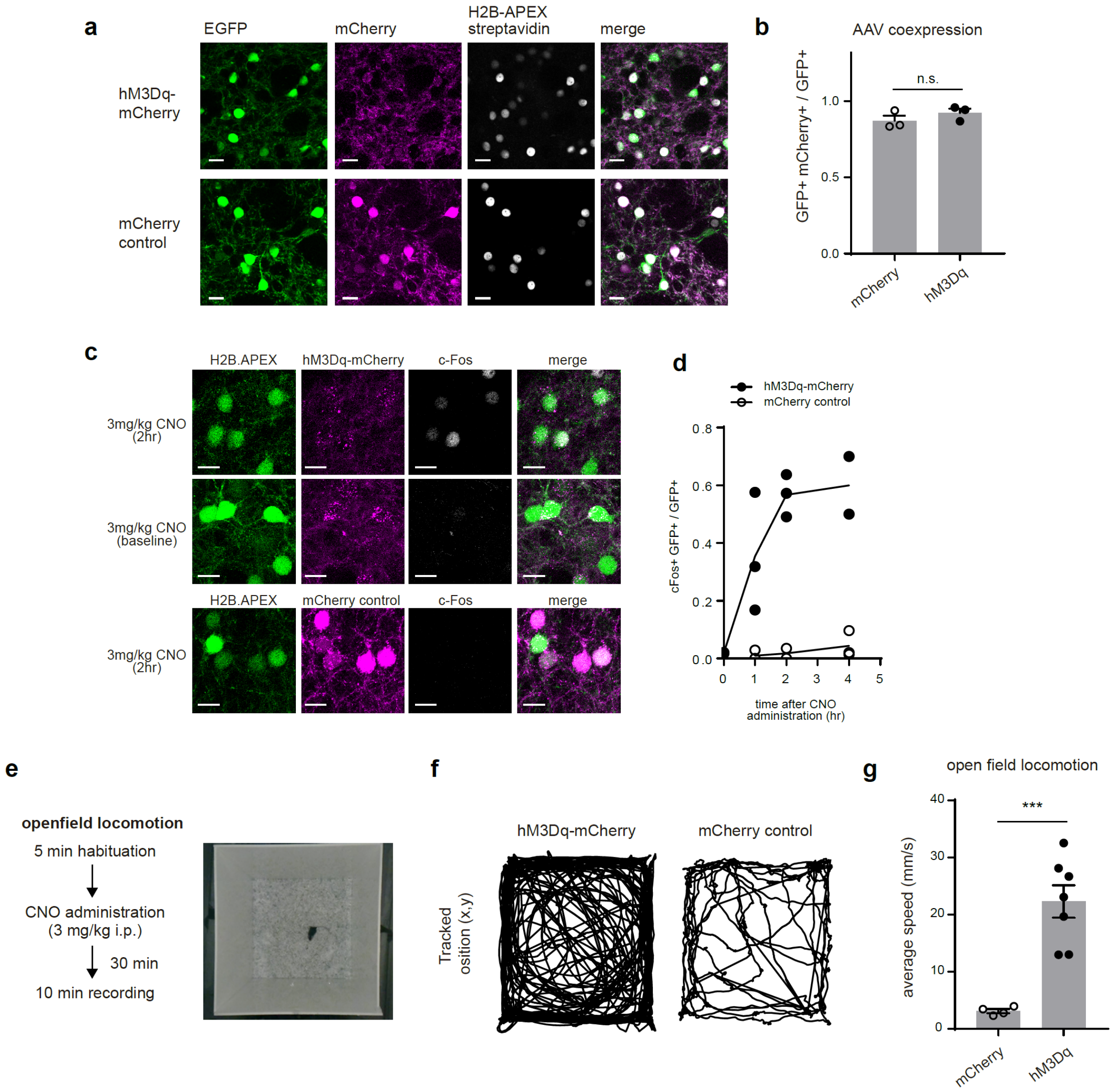

Supplementary Figure 9. Validation of chemogenetic activation paradigm.

(a) H2B.APEX and hM3Dq co-expression in striatal Drd1^{Cre} neurons. Scale bar: 20 μm.
(b) Quantification of APEX and hM3Dq co-expression. Total 393 and 433 GFP+ cells from n = 3 animals for mCherry and hM3Dq, respectively. Mann-Whitney test, p = 0.20. Error bars reflect SEM.
(c) *In vivo* administration of Clozapine-N-oxide (CNO) selectively activates hM3Dq-expression neurons, indicated by *c-Fos* expression. Scale bar: 20 μm.
(d) Quantification of (c). *c-Fos* expression time course after CNO administration. For hM3Dq: 212, 330, 355, 220 GFP+ cells from n = 2, 3, 3, 2 animals per time point. For mCherry control: 301, 211, 282 GFP+ cells from n = 3, 2, 3 animals per time point.
(e) Open-field location assay.
(f) Activation of striatal dSPNs increases animal locomotor activity. Representative traces for 10 min locomotion tracking.
(g) Quantification of animal locomotor activity 30 min post CNO administration. n = 4, 7 animals for mCherry control and hM3Dq, respectively. Unpaired two-tailed t-test, p = 0.0008. Error bars reflect SEM. Source data are provided as a Source Data file.

a

TMT 11plex experimental design

	channel	126	127N	127C	128N	128C	129N	129C	130N	130C	131N	131C
set 1	Condition	hM3Dq	RFP	hM3Dq	Cre-/-	NES	RFP	hM3Dq	RFP	NES	Cre-/-	REF
	APEX	H2B	H2B	H2B	None	NES	H2B	H2B	H2B	NES	None	H2B
	replicate#	1	1	4	1	1	4	7	3	3	3	

	channel	126	127N	127C	128N	128C	129N	129C	130N	130C	131N	131C
set 2	Condition	RFP	hM3Dq	Cre-/-	NES	hM3Dq	RFP	RFP	hM3Dq	hM3Dq	RFP	REF
	APEX	H2B	H2B	None	NES	H2B	H2B	H2B	H2B	H2B	H2B	H2B
	replicate#	7	2	2	2	5	2	5	6	3	6	

condition	No. of replicates
H2B.hM3Dq	7
H2B.RFP	7
NES	3
Cre-/- no APEX	3
REF Ch.	2

b

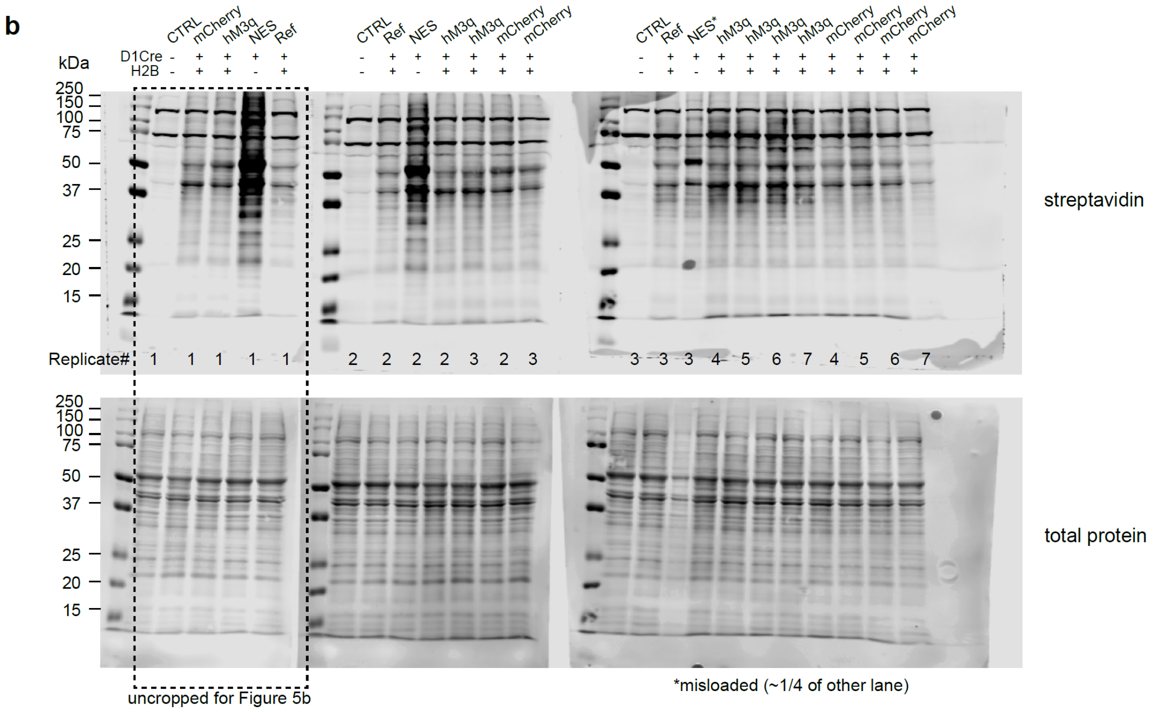

c

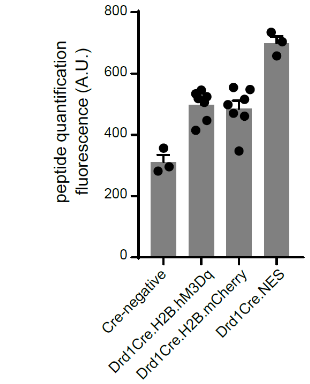

Supplementary Figure 10. Experimental design and sample quality check for the chemogenetic experiment.

(a) TMT 11plex experimental design.
(b) Western blot analysis of biotinylated lysates before MS sample preparation. Dotted line shows cropped region for blot in Fig 5b. Western blot analysis of these samples was performed once prior to orthogonal confirmation by proteomics.
(c) Relative peptide output after on-bead digestion before TMT labeling. Cre-negative n = 3, Drd1^{Cre}.H2B.hM3Dq n = 7, Drd1^{Cre}.H2B.mCherry n = 7, and Drd1^{Cre}.NES n = 3 animals/sample. Error bars reflect SEM. Source data are provided as a Source Data file.

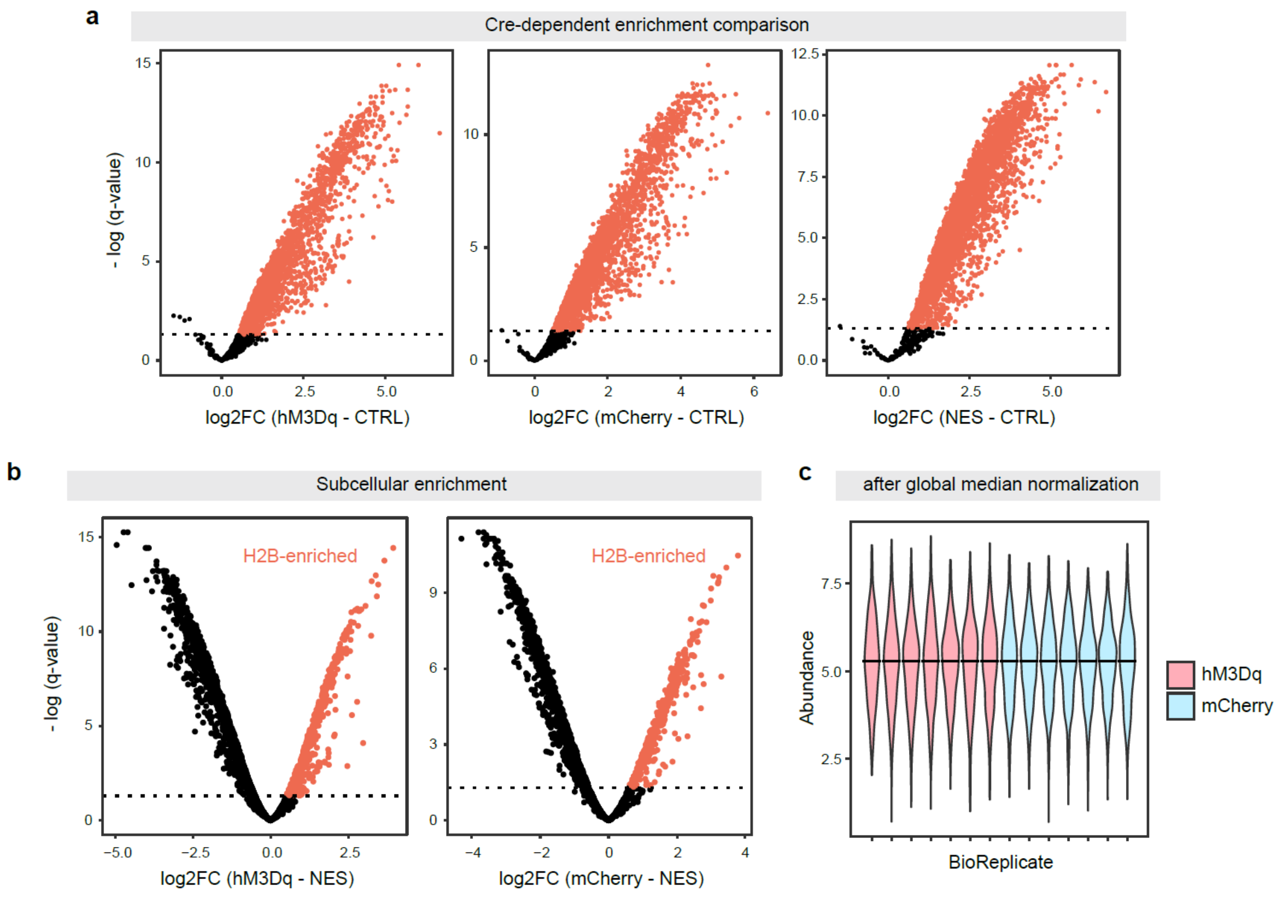

Supplementary Figure 11. Nuclear proteome cutoff analysis for the DREADD experiment.

(a) Cre-dependent enrichment of H2B.hM3Dq, H2B.mCherry, and APEX.NES over Cre-negative control.
(b) H2B-NES comparison for H2B.hM3Dq or H2B.mCherry. Red dots indicate H2B-enriched proteins.
(c) Overall protein intensities across biological replicates after protein-level median normalization. Source data are provided as a Source Data file.

a

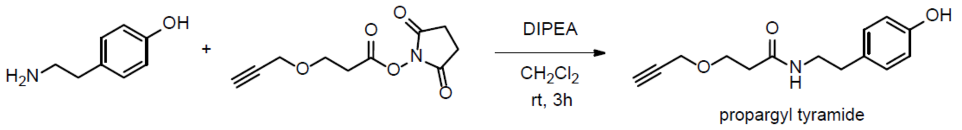

b

^{1}H NMR (500 MHz, Chloroform-d) δ 7.09 – 6.97 (m, 2H), 6.85 – 6.68 (m, 2H), 6.13 (m, 2H), 4.08 (d, J = 2.4 Hz, 2H), 3.71 (t, J = 5.7 Hz, 2H), 3.48 (td, J = 7.0, 5.8 Hz, 2H), 2.72 (t, J = 6.9 Hz, 2H), 2.54 – 2.32 (m, 3H).

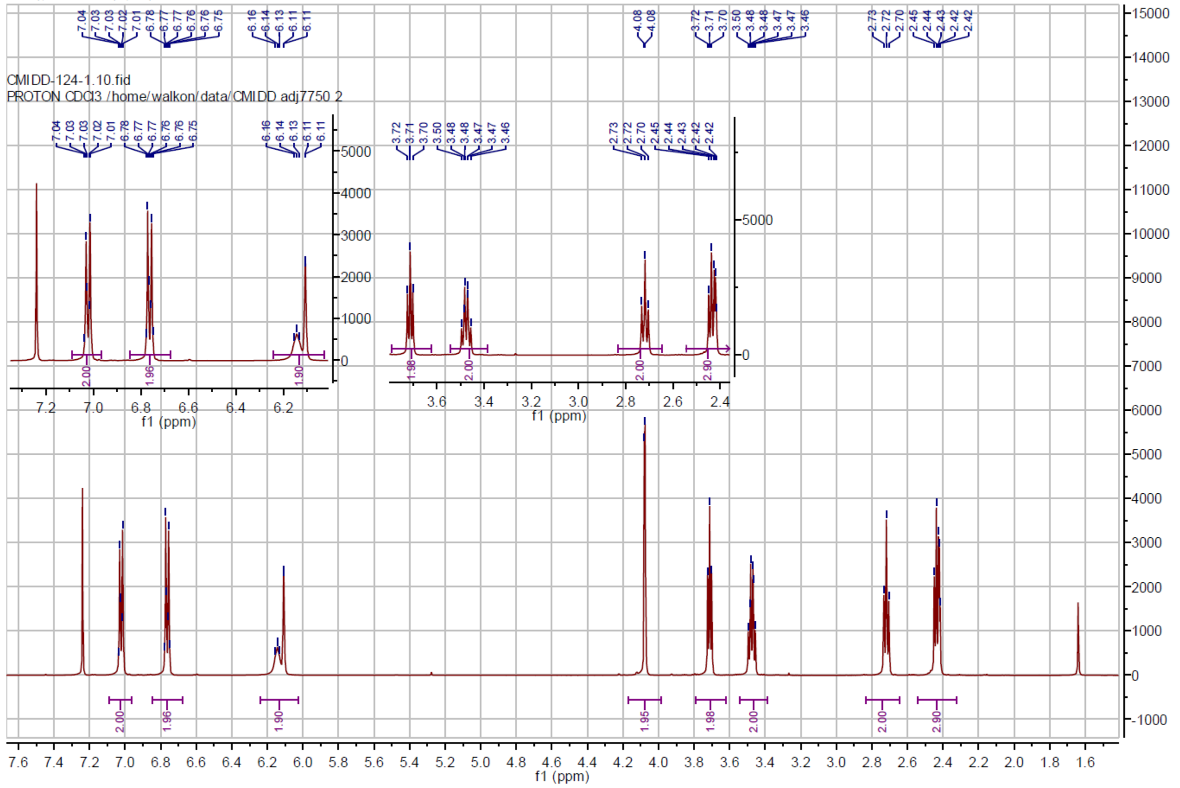

c

^{13}C NMR (126 MHz, Chloroform-d) δ 171.45, 154.74, 130.38, 129.83, 115.51, 79.10, 75.02, 65.98, 58.33, 40.81, 36.89, 34.66.

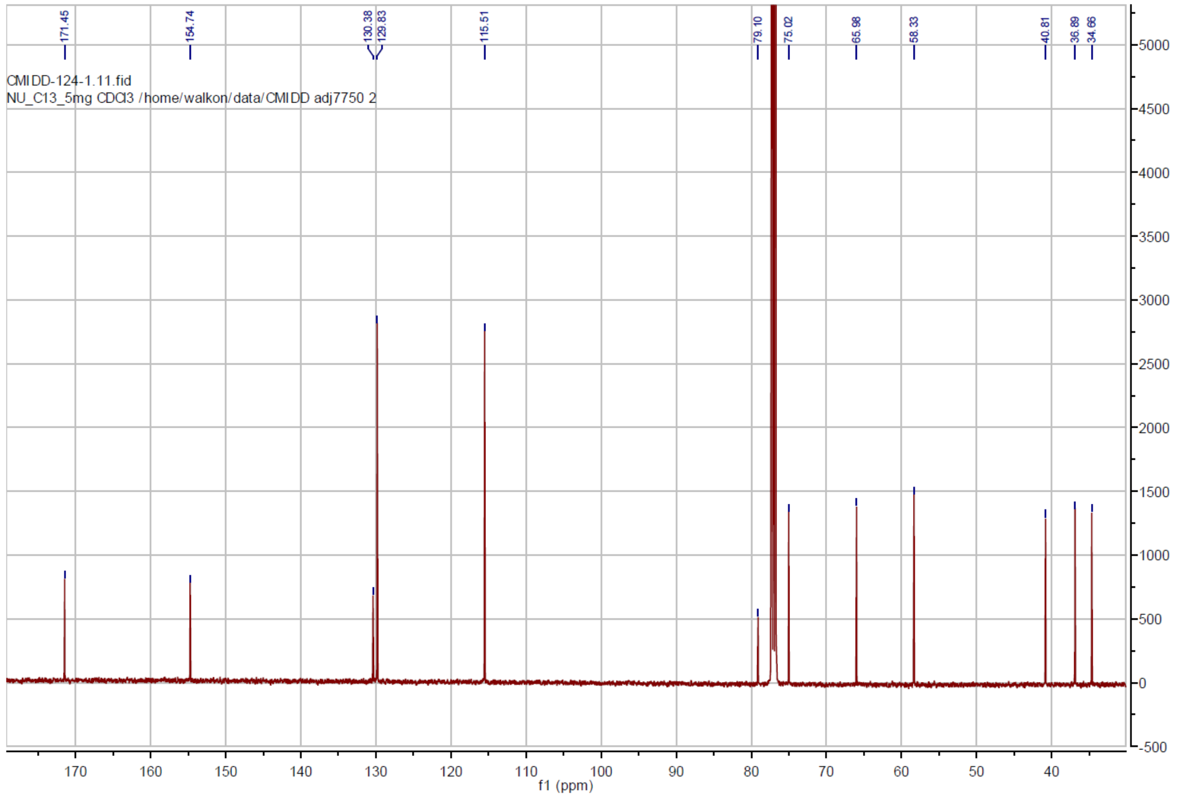

Supplementary Figure 12. Synthesis of propargyl tyramide.

(a) Synthesis of propargyl tyramide.
(b) ^{1}H-NMR of propargyl tyramide.
(c) ^{13}C-NMR of propargyl tyramide.

Supplementary Table 1. Custom liquid chromatography gradients for high pH reverse-phase (HpHRP) fractionation samples.

	Time (min)					
HpHRP Fraction (% ACN)	0	1	5	10	160	180
5	2	2	5		17.5	45
10	2	2	5		20	45
12.5	2	2	5		22	45
15	2	2	5		25	45
17.5	2	2		8	25	45
20	2	2		8	25	45
22.5	2	2		10	25	45
25	2	2		10	25	45
50	2	2		10	25	45

Number indicates %B
