# Supplementary material for: Cell-type and subcellular compartment-specific APEX2 proximity labeling reveals activity-dependent nuclear proteome dynamics in the striatum
Source: Nat Commun. 2021 Aug 11;12:4855. doi: 10.1038/s41467-021-25144-y (PMC8357913; doi:10.1038/s41467-021-25144-y)
Supplement: Supplementary file 3 — Description of Additional Supplementary Files [file 41467_2021_25144_MOESM3_ESM.pdf]

Description of Additional Supplementary Files

Supplementary Data 1. Protein identification and summary for data in Fig. 2-3 (tab 1: Protein identification without fractionation; tab 2: Protein identification with fractionation; tab 3: Protein quantification by MSstatsTMT for samples with fractionation).

Supplementary Data 2. Statistical comparison for data in Fig. 2-3. Unpaired two-sided moderated t-tests. p-values and Benjamini-Hochberg adjusted p-values are included (tab 1: APEX.NES vs CTRL; tab 2: H2B.APEX vs CTRL; tab 3: LCK.APEX vs CTRL; tab 4: H2B.APEX vs APEX.NES; tab 5: H2B.APEX vs LCK.APEX; tab 6: LCK.APEX vs APEX.NES; tab 7: a list of H2B-enriched proteins in $Drd1^{Cre+}$ dSPNs; tab 8: a list of LCK-enriched proteins in $Drd1^{Cre+}$ dSPNs).

Supplementary Data 3. DAVID gene ontology analysis for H2B-enriched nuclear proteins and LCK-enriched membrane proteins for data in Fig. 2-3.

Supplementary Data 4. Protein identification and summary for data in Fig. 4 (tab 1: Protein identification; tab 2: The contaminant list for filtering out highly abundant non-specific binders and astrocyte-enriched proteins; tab 3: Protein quantification by MSstatsTMT).

Supplementary Data 5. Statistical comparison for data in Fig. 4. Unpaired two-sided moderated t-tests. p-values and Benjamini-Hochberg adjusted p-values are included. (tab 1: H2B.APEX vs APEX.NES for $Drd1^{Cre+}$ dSPNs; tab 2: H2B.APEX vs APEX.NES for $A2a^{Cre+}$ iSPNs; tab 3: H2B-enriched nuclear proteins; tab 4: non-H2B enriched cytosolic proteins; tab 5: H2B-enriched nuclear comparison (iSPN - dSPN); tab 6: non-H2B enriched cytosolic protein comparison (iSPN - dSPN)).

Supplementary Data 6. DAVID gene ontology analysis for H2B-enriched nuclear proteins for data in Fig. 2-3.

Supplementary Data 7. Protein identification and summary for data in Fig. 5 (tab 1: Protein identification; tab 2: Protein quantification by MSstatsTMT for nuclear proteins; tab 3: for cytosolic proteins).

Supplementary Data 8. Statistical comparison for data in Fig. 5. Unpaired two-sided moderated t-tests. p-values and Benjamini-Hochberg adjusted p-values are included. (tab 1: APEX.NES vs CTRL; tab 2: H2B.APEX_mCherry vs CTRL; tab 3: H2B.APEX_hM3Dq vs CTRL; tab 4: H2B.APEX_mCherry vs APEX.NES; tab 5: H2B.APEX_hM3Dq vs APEX.NES; tab 6: H2B enriched nuclear protein comparison (hM3Dq – mCherry); tab 7: non-H2B enriched cytosolic protein comparison (hM3Dq – mCherry)).

Supplementary Data 9. DAVID gene ontology analysis for H2B-enriched proteins for data in Fig. 5.

Supplementary Data 10. Differentially regulated transcripts and proteins for the correlation analysis in Fig. 4.
