# Supplementary material for: Cell-type and subcellular compartment-specific APEX2 proximity labeling reveals activity-dependent nuclear proteome dynamics in the striatum
Source: Nat Commun. 2021 Aug 11;12:4855. doi: 10.1038/s41467-021-25144-y (PMC8357913; doi:10.1038/s41467-021-25144-y)
Supplement: Supplementary file 14 — Reporting Summary [file 41467_2021_25144_MOESM14_ESM.pdf]

nature research

Corresponding author(s): MacDonald, Kozorovitskiy
Last updated by author(s): 7/22/2021

Reporting Summary

Nature Research wishes to improve the reproducibility of the work that we publish. This form provides structure for consistency and transparency in reporting. For further information on Nature Research policies, see our Editorial Policies and the Editorial Policy Checklist.

Statistics

For all statistical analyses, confirm that the following items are present in the figure legend, table legend, main text, or Methods section.

n/a	Confirmed	
☐	☒	The exact sample size (*n*) for each experimental group/condition, given as a discrete number and unit of measurement
☐	☒	A statement on whether measurements were taken from distinct samples or whether the same sample was measured repeatedly
☐	☒	The statistical test(s) used AND whether they are one- or two-sided<br>*Only common tests should be described solely by name; describe more complex techniques in the Methods section.*
☐	☒	A description of all covariates tested
☐	☒	A description of any assumptions or corrections, such as tests of normality and adjustment for multiple comparisons
☐	☒	A full description of the statistical parameters including central tendency (e.g. means) or other basic estimates (e.g. regression coefficient) AND variation (e.g. standard deviation) or associated estimates of uncertainty (e.g. confidence intervals)
☐	☒	For null hypothesis testing, the test statistic (e.g. *F*, *t*, *r*) with confidence intervals, effect sizes, degrees of freedom and *P* value noted<br>*Give P values as exact values whenever suitable.*
☒	☐	For Bayesian analysis, information on the choice of priors and Markov chain Monte Carlo settings
☒	☐	For hierarchical and complex designs, identification of the appropriate level for tests and full reporting of outcomes
☐	☒	Estimates of effect sizes (e.g. Cohen's *d*, Pearson's *r*), indicating how they were calculated

Our web collection on statistics for biologists contains articles on many of the points above.

Software and code

Policy information about availability of computer code

Data collection	Raw MS data were collected using a Thermo Scientific Orbitrap Eclipse mass spectrometer operated in MS3 with real-time search. Detailed description can be found in the method section.<br>A modified version of Scanimage version 3.6 (Pologruto et al, 2003) was used for electrophysiology experiments.<br>Transmission Electron Microscope (TEM) images were collected using Digital Micrograph software (version 1.82.305).
Data analysis	Group statistical analyses (except proteomics data) were done using GraphPad Prism 7 software (GraphPad, LaJolla, CA).<br>All imaging data were analyzed using FIJI version 1.53c (Schindelin et al, 2012).<br>Analysis of electrophysiology data was performed using Igor Pro version 6.1 (Wavemetrics, Portland, OR) and MATLAB 2017 (Mathworks, Natick, MA).<br>Western blot analysis was performed in Image Studio version 5.2 (LI-COR).<br>Raw MS files were processed in Proteome Discoverer version 2.4 (Thermo Scientific, Waltham, MA).<br>Statistical analysis of proteomics data was done in R (version 4.0.5) and RStudio (version 1.3.1093) using MSstatsTMT package (version 1.7.3) (Huang et. al., 2020). All parameters were included in the method section. All computer code used in this study is posted to the laboratory GitHub account (https://github.com/KozorovitskiyLaboratory/proteomics_APEX).<br>Open field locomotion behavior was analyzed by Toxtrac (Version 2.90) (Rodriguez, A., et al., 2018).<br>Network analysis were constructed in Cytoscape version 3.8.2.

For manuscripts utilizing custom algorithms or software that are central to the research but not yet described in published literature, software must be made available to editors and reviewers. We strongly encourage code deposition in a community repository (e.g. GitHub). See the Nature Research guidelines for submitting code & software for further information.

Data

Policy information about availability of data

All manuscripts must include a data availability statement. This statement should provide the following information, where applicable:

- Accession codes, unique identifiers, or web links for publicly available datasets
- A list of figures that have associated raw data
- A description of any restrictions on data availability

The raw MS data generated in this study have been deposited in the PRIDE database under accession code PXD022335 [https://www.ebi.ac.uk/pride/archive/projects/PXD022335]. Analyzed data generated in this study are provided in the Supplementary Information and Source Data file. The reference number for the mouse SwissProt database used in this study is 000000589 [https://www.uniprot.org/uniprot/?query=proteome:UP000000589%20reviewed:yes]. For network analysis, two online databases were used including STRING-DB v11 [https://string-db.org/] and HuRI [http://www.interactome-atlas.org/]. All datasets and plasmids generated in this study are available from the corresponding author on reasonable request. Source data are provided.

Field-specific reporting

Please select the one below that is the best fit for your research. If you are not sure, read the appropriate sections before making your selection.

☒ Life sciences ☐ Behavioural & social sciences ☐ Ecological, evolutionary & environmental sciences

For a reference copy of the document with all sections, see nature.com/documents/nr-reporting-summary-flat.pdf

Life sciences study design

All studies must disclose on these points even when the disclosure is negative.

Sample size	Required sample sizes were estimated based on previous publications (Rhee et al Science 2013, Loh et al Cell 2016, Loingier et al Cell 2017, and Paek et al Cell 2017) and our past experience.
Data exclusions	No data were excluded after analyses.
Replication	The number of replicates for each experiment were reported in the paper, figure legends, and the method section. Each proteomic dataset was performed once, while the biological replicates were collected from multiple independent tissue preparation sessions as animals become available. All attempts at replication were successful. All other experiments were repeated at least twice or as noted in the figure legend.
Randomization	Animals were randomly assigned to groups. Proteomic samples were randomized for TMT labeling experiments.
Blinding	Whenever possible, data were analyzed blind to condition. Data that require an automated analysis pipeline were not analyzed blind to conditions, including proteomics data (MSstatsTMT), locomotion behavior (ToxTrac). For cFos analysis, the investigators were blinded to group assignment during cell counting.

Reporting for specific materials, systems and methods

We require information from authors about some types of materials, experimental systems and methods used in many studies. Here, indicate whether each material, system or method listed is relevant to your study. If you are not sure if a list item applies to your research, read the appropriate section before selecting a response.

Materials & experimental systems

n/a	Involved in the study	
☐	☒	Antibodies
☐	☒	Eukaryotic cell lines
☒	☐	Palaeontology and archaeology
☐	☒	Animals and other organisms
☒	☐	Human research participants
☒	☐	Clinical data
☒	☐	Dual use research of concern

Methods

n/a	Involved in the study	
☒	☐	ChIP-seq
☒	☐	Flow cytometry
☒	☐	MRI-based neuroimaging

Antibodies

Antibodies used	Primary antibodies used in the study were listed below:<br>mouse anti-FLAG (1:1000; Cat. No. A00187-200, Genscript, NJ, USA)<br>chicken anti-GFP (1:2000; Cat. No. AB13970, Abcam, Cambridge, UK)<br>rabbit anti-c-Fos (1:10,000; Cat. No. 226003, Synaptic Systems, Goettingen, Germany)<br>rabbit anti-JunB (C37F9) (1:5,000) (Cat. No. 3753S, Cell Signaling, Danvers, MA)

	All secondary antibody staining steps were performed at 1:500 dilution. Secondary antibodies for immunofluorescence staining are listed below (ThermoFisher):<br>goat anti-mouse Alexa 594 (Cat. No. A11032)<br>goat anti-rabbit Alexa 647 (Cat. No. A11008)<br>goat anti-chicken Alexa 488 (Cat. No. A11039)<br>streptavidin Alexa 647 (Cat. No. S32357)<br><br>All secondary antibody/detection reagents for western blotting are listed below:<br>streptavidin CW800 (Cat. No. 926-32230, LICOR, Lincoln, NE, USA)
Validation	All antibodies are commercially available and validated in multiple studies. Complete information is available in the data sheets on the manufacturer's website.<br><br>mouse anti-FLAG (1:1000; Cat. No. A00187-200, Genscript, NJ, USA) – the antibody was validated against cell lysates with or without FLAG-tagged proteins.<br>chicken anti-GFP (1:2000; Cat. No. AB13970, Abcam, Cambridge, UK) – the antibody was validated against tissues with GFP, without GFP, with RFP (mCherry and tdTomato) and with both GFP and RFP. The antibody shows specificity for GFP, but not RFP.<br>rabbit anti-c-Fos (1:10,000; Cat. No. 226003, Synaptic Systems, Goettingen, Germany) – the antibody has been used and validated in our lab (Wu et. al. eLife 2021;10:e64041, DOI: 10.7554/eLife.64041)<br>rabbit anti-JunB (C37F9) (1:5,000) (Cat. No. 3753S, Cell Signaling, Danvers, MA) – the antibody was validated in multiple cell lines and human lung carcinoma tissues with or without control peptides.

Eukaryotic cell lines

Policy information about cell lines

Cell line source(s)	HEK293T line was obtained from ATCC (293T/17 [HEK 293T/17], CRL-11268).
Authentication	No authentication was performed.
Mycoplasma contamination	Not tested for mycoplasma contamination.
Commonly misidentified lines (See ICLAC register)	None. HEK293T was not listed in the ICLAC.

Animals and other organisms

Policy information about studies involving animals; ARRIVE guidelines recommended for reporting animal research

Laboratory animals	Weanling and young adult male and female mice were used in this study. Drd1Cre (262Gsat/Mmcd) and A2aCre(KG139Gsat) was obtained from Mutant Mouse Regional Resource Center (MMRRC) at the University of California, Davis. C57BL/6 mice used for breeding and backcrossing were acquired from Charles River (Wilmington, MA). All mice were group-housed in a humidity-controlled, ambient temperature facility, with standard feeding, 12hr light-dark cycle, and enrichment procedures; littermates were randomly assigned to conditions. All animals were genotyped according to the MMRRC strain-specific primers. P35-70 male and female mice were used in the experiments.
Wild animals	No wild animals were used in the study.
Field-collected samples	No field collected samples were used in the study.
Ethics oversight	Animals were handled according to protocols approved by the Northwestern University Animal Care and Use Committee.

Note that full information on the approval of the study protocol must also be provided in the manuscript.
